# Novel one-step lignin microsphere preparation for oral tissue regeneration applications

**DOI:** 10.3389/fbioe.2024.1521223

**Published:** 2025-01-07

**Authors:** Anyuan Shi, Li Guo, Chunning Gu, Yunni Zhan, Xuelian Zhou, Wei Cheng

**Affiliations:** ^1^ Department of Dental Implantology, Nanjing Stomatological Hospital, Affiliated Hospital of Medical School, Institute of Stomatology, Nanjing University, Nanjing, China; ^2^ Institute of Chemical Industry of Forest Products, Chinese Academy of Forestry, Nanjing, China

**Keywords:** deep eutectic solvent, lignin microspheres, antioxidant activity, *in vitro* biomedical test, biocompatibility

## Abstract

Lignin is a naturally derived biomacromolecule with excellent biocompatibility and the potential for biomedical application. For the first time, this study isolated nanosized lignin microspheres (LMSs) directly from wheat straw with a polyol-based deep eutectic solvent. The size of these LMSs can be regulated by changing the isolation parameters, ranging from 90 nm to 330 nm. The structures of these LMSs were comprehensively investigated by SEM, gel permeation chromatography (GPC), HSQC NMR, and ^31^P NMR, which explained the formation mechanism of the hydrophobicity-induced self-assembly. The LMSs show good antioxidation of 52.99%–76.26% toward DPPH. *In vitro* biomedical tests further revealed that the LMSs at concentrations <25 μg/mL had good biocompatibility toward gingival mesenchymal stem cells (GMSCs) and jaw bone marrow mesenchymal stem cells (JBMMSCs), with a low apoptosis rate, outperforming other lignin materials. The presented results highlighted the application of the nanosized LMSs as a potential biomaterial in oral tissue regeneration.

## 1 Introduction

As the major component of lignocellulose, lignin is the glue that combines cellulose and hemicellulose with various chemical and physical bonds, endowing the mechanical strength of plants (Meng et al., 2020). Generally, lignin is a linear macromolecule made up of three basic units of syringyl, guaiacyl, and *p*-hydroxyphenyl, which are covalently linked by C-O (*e.g.*, β-O-4 and α-O-4) and C-C (*e.g.*, β-β and β-5) bonds (Bai et al., 2022). Currently, 50–70 million tons of lignin is produced annually, mostly in the pulping process (Bajwa et al., 2019). The kraft process, the main pulping technology used worldwide, uses NaOH and NaS_2_ as the delignification regents (Crestini et al., 2017). This process is normally accomplished at a temperature >160°C for 3–4 h, which causes significant delignification by the disruption of lignin interlinkages. The kraft lignin can be readily recovered by acid precipitation with a high yield (>90%) (Chakar and Ragauskas, 2004). In addition, the sulfite process is also a popular method that sulfonates lignin and renders a soluble lignin salt termed lignosulfonate. However, the isolation and purification of the lignosulfonate may need tedious steps as it is water-soluble, in contrast to the kraft lignin.

In recent decades, biorefining that aims to isolate the three major components (cellulose, hemicellulose, and lignin) in biomass has attracted significant attention. These technologies include organosolv, ionic liquids, deep eutectic solvents, and so forth. The resulting lignins have their own functionality and special structure, rendering wide applications (Lynd et al., 2022). In all these isolation methods, lignin simultaneously suffers from degradation and condensation, and the condensation reaction tends to yield lignin with stubborn C-C linkages that are difficult to valorize (Renders et al., 2017).

Lignin applications have been extensively researched for their abundance, specific aromatic structure, and phenolic hydroxyl groups in the aspects of adhesive, phenolic compounds, carbon nanofiber, and others (Kai et al., 2016). In the most recent decade, lignin has been widely used as a biomedical material due to its excellent antioxidation, anti-inflammatory, and even antitumor effects (Alqahtani et al., 2019). These effects are caused by the abundance of phenols and polyphenols in lignin that could effectively eliminate potential radical oxygen species (hydrogen peroxide, superoxide anions, and hydroxyl radicals), hindering the proliferation of bacteria and enhancing the human immune system (Pei et al., 2023). Previous studies indicated that lignin strongly inhibits viruses, such as human immunodeficiency virus and herpes simplex virus. Its mechanism is quite different from that of bacteria, including inactivation of the cellular entry of the virus and damage to adsorption of the virus to human cells (Liu et al., 2022). Moreover, lignin shows excellent biocompatibility as it is directly isolated from natural biomass, which favors its potential in biomedical applications.

Due to the abovementioned properties, lignin has wide applications as a gradient in tissue engineering, aiming to enhance mechanical strength and cell affinity. This facilitates the use of lignin to fabricate various composites that can be applied in various biomedical applications. Wang et al. reported a lignin/polycaprolactone scaffold that can effectively induce the precipitation of hydroxyapatite and further facilitate the adhesion and proliferation of osteoblasts (Wang et al., 2019). Deng et al. proposed a lignin-based hydrogel having excellent blood compatibility and broad-spectrum antimicrobial and tissue adhesion properties, which *in vivo* accelerated the hemostasis of a rat liver injury (Deng et al., 2021). Lignin-based film, aerogel, fiber, and other materials were also reported to have good performance in liver/kidney regeneration, neural therapy, and wound healing (Wang et al., 2022; Sato et al., 2020; Saudi et al., 2019). Almost all these studies used kraft lignin as the starting material, which requires tedious purification, dialysis, and fractionation to obtain the target lignin fraction for the following research. These problems render lignin’s biomedical application in its infancy and far from scale-up utilization.

Lignin microspheres (LMS) are a kind of new nano/micro lignin materials. They can generally be obtained by the antisolvent technique in which lignin is first dissolved in a solvent (ethanol, ethylene glycol, alkali, and others) and then dialyzed in pure water. Liu et al. produced LMSs using acetone solution as a solvent, which yielded an LMS with the smallest size of 142 nm (Liu et al., 2018). In another study, Qian et al. modified alkali lignin by acetylation and then dissolved the sample in THF for the LMS self-assembly in the following water–solvent exchange. This method resulted in an LMS with a diameter of 110 nm (Qian et al., 2014). In these studies, LMSs are generated with the hydrophobicity of the lignin (from both the phenolic OH and the aromatic ring) that formed a stable lignin nucleus and further facilitated the growth of the LMSs. Lignin has also been applied in biomedical areas as it has a very specific surface area and can benefit cell attachment and proliferation (Figueiredo et al., 2018). However, the traditional LMS method usually has a very low yield (<10%), a complicated preparation process, and very high cost, and is far from scale-up application.

Oral diseases are often associated with bacterial infection and inflammation due to dental caries, periodontitis, mucous membrane lesions, oral cancer, preimplant inflammation, temporomandibular arthritis, orthodontic bone resorption, etc. (D'Aiuto et al., 2017). Thus, there is a great clinical need for materials with anti-inflammatory and regeneration functions in stomatology. In this study, we proposed a new method that could isolate lignins from lignocellulose and then directly transform them into LMSs in a one-pot process. The LMS size was determined by the pretreatment condition and was in the range of 90–330 nm. Next, these LMSs were investigated for oral repair applications by *in vitro* cultivation with the gingival mesenchymal stem cells (GMSCs) and jaw bone marrow mesenchymal stem cells (JBMMSCs), which showed good biocompatibility and low cytotoxicity. This research highlights our recent study in the aspects of both LMS generation and their biomedical application and may open a new door for further lignin scale-up applications.

## 2 Materials and methods

### 2.1 Materials

Wheat straw (WS) was obtained from a local farm in Jiangsu Province, China, chopped, and then milled into powder before lignin isolation. Choline chloride (ChCl), 1,4-butanediol (BDO), and anhydrous AlCl_3_ were provided by Sinopharm Chemicals (Beijing, China). Modified minimum essential medium (α-MEM) was purchased from Gibco, Grand Island, NY, United States. Fetal bovine serum, L-ascorbic acid 2-phosphate, 1% penicillin/streptomycin, and trypan blue solution were all from Sigma-Aldrich, St. Louis, MO, United States.

### 2.2 Deep eutectic solvent (DES) synthesis and lignin isolation

The DES was synthesized by blending ChCl, BDO, and anhydrous AlCl_3_ at a molar ratio of 25:50:1 in a glass bottle at 80°C for 1 h until yielding transparent and clear liquid. It was then stored in a desiccator before use.

For lignin isolation, a one-factor-at-time method, which is widely adopted in the optimization of biomass pretreatment, was used to investigate how temperature and time affect efficiency. In detail, 10 g (dry weight) wheat straw was first mixed with 100 g DES, which was then placed in a preheated oil bath at 90°C–140°C for 1–6 h with mechanical agitation. Upon the completion of pretreatment, 500 mL acetone solution (50 v/v%) was added to quench the reaction, followed by stirring at RT for 2 h. Then, the pretreatment solid and liquid were separated by vacuum filtration. The pretreated solid was washed with an additional 200 mL of acetone/water (1:1 volume ratio) solution to separate residual lignin, after which it was combined with the pretreatment liquid. The total pretreatment liquid was then subjected to rotary evaporation to remove the acetone. Then, 1 L of DI water was added to precipitate the lignin. Finally, the precipitated lignin was obtained by centrifugation and then freeze-dried and stored at 4°C (Cheng et al., 2023a).

### 2.3 Characterization methods

Cellulolytic enzyme lignin (CEL) in raw WS was isolated using sequential ball milling, enzymatic hydrolysis, and dioxane extraction. In detail, the WS was first subjected to ball milling for 24 h. After that, the WS powder was subjected to enzymatic hydrolysis using *Novozymes Ctec3* at 100 FPU/g-cellulose for 48 h. The enzymatic hydrolysis was repeated two times to ensure sufficient removal of carbohydrates. Next, the residue after enzymatic hydrolysis was freeze-dried and then extracted using 96 v/v% dioxane. Finally, the CEL was obtained by rotary evaporation, water washing, and freeze-drying.

Composition analysis of the solid and LMSs was performed following the NREL two-step sulfuric acid hydrolysis protocols, as detailed in our previous publication (Zhan et al., 2022). Monomeric sugars from the acid hydrolysis were measured with HPLC (high-performance liquid chromatography). The equation for the lignin removal is shown below.

$$Lignin\;removal\;\left(\%\right)=\frac{residual\;lignin\;in\;pretreated\;biomass\;\left(g\right)}{lignin\;in\;raw\;biomass}\times 100\%.$$

The micromorphology of the LMSs was observed with a Hitachi field emission SEM (FE-SEM) at a 10 kV voltage. Before the test, the LMSs were first dried in a 50°C oven overnight and then sputter-coated with gold for 10 s. The size distribution (D50) of the LMSs was analyzed using a zeta sizer (Nano ZS, Malvern Instrument, United Kingdom). For the test, LMSs were dispersed in DI water at a concentration of 0.01%, and the mixture was then subjected to ultrasound treatment to further disperse the LMSs.

The molecular weight of the LMSs was determined by gel permeation chromatography (GPC). LMSs were first acetylated to increase their solubility by reaction in the pyridine/acetic anhydride for 24 h in the dark. After that, the acetylated lignin (freeze-dried sample) was redissolved in tetrahydrofuran at a concentration of 2 g/L and then subjected to the GPC test using polystyrene as the inner standard.

The chemical structure of the LMSs was analyzed by the HSQC NMR using a Bruker Ascend™ 600 MHz instrument. For the sample preparation, ∼150 mg of dried LMSs were first dissolved in 0.6 mL DMSO-*d*6 and then subjected to ultrasound treatment until it was totally dissolved. The detailed acquisition parameters can be found in our previous publication (Huang et al., 2017). Various hydroxyl groups in the raw WS CEL and regenerated LMSs were measured using ^31^P NMR. The detailed preparation and determination operations can be found in the previous publication (Cheng et al., 2023b).

### 2.4 Cell culture

Human gingival mesenchymal stem cells (GMSCs) and jaw bone marrow mesenchymal stem cells (JBMMSCs) derived from soft and hard oral tissues were used in this study. The isolation and culture methods were adopted, as referred to our previous publication (Shi et al., 2019). These cells were cultured using a medium containing α-modified minimum essential medium (α-MEM) supplemented with 15% fetal bovine serum, 100 mM L-ascorbic acid 2-phosphate, and 1% penicillin/streptomycin in a humidified incubator at 37°C and 5% CO_2_. The culture medium was changed every 3 days. Cells at passage 5 were used for the subsequent experiments.

### 2.5 Apoptosis assay

To examine the effect of lignin treatment on the apoptosis of GMSCs and JBMMSCs, approximately 1×10^5^ cells were cultured in a 35-mm culture plate for this assay. When reaching 60%–70% confluency, the cells were incubated with different concentrations of lignin (0 μg/mL, 1 μg/mL, 10 μg/mL, 25 μg/mL, 50 μg/mL, 100 μg/mL, and 200 μg/mL) for 24 h. Then, the single-cell suspension was stained following the manufacturer’s protocol of the ANNEXIN V-FITC/PI Apoptosis Detection Kit (Solarbio, Beijing, China). Rates of apoptosis were analyzed using the FACSCalibur system (BD Biosciences, Mountain View, CA). Five parallel replicates of each sample at each time point were prepared during this assay. Experiments were performed in triplicate for each group.

### 2.6 Cell viability assay

To evaluate the cytotoxicity of lignin to GMSCs and JBMMSCs, approximately 3.5×10^5^ cells were cultured in a 60-mm culture plate for cell viability assay. When reaching 60%–70% confluency, the cells were treated with different concentrations of lignin (0 μg/mL, 1 μg/mL, 10 μg/mL, 25 μg/mL, 50 μg/mL, 100 μg/mL, and 200 μg/mL) for 48 h, respectively. Then, the detached single-cell suspension was stained with 0.4% Trypan blue solution, and the live cells were counted by a cell counter (RWD C100, Shenzhen, China). Five parallel replicates of each sample at each time point were prepared during this assay. Experiments were performed in triplicate for each group.

### 2.7 Cell growth assay

The proliferation of cells treated with lignin was analyzed using the enhanced Cell Counting Kit-8 (CCK-8; Beyotime, Shanghai, China). Approximately 2×10^3^ cells suspended in medium (200 μL) were seeded in 96-well plates with lignin (0 μg/mL, 1 μg/mL, 10 μg/mL, 25 μg/mL, 50 μg/mL, 100 μg/mL, and 200 μg/mL) and incubated for different time intervals (1 day, 2 days, 3 days, 4 days, 5 days, and 6 days). CCK-8 solution (20 μL) was added to each well. The cultures were incubated at 37°C for 2 h. Absorbance at a wavelength of 450 nm was measured by a SpectraMax M3 microplate reader (Molecular Devices, Sunnyvale, USA). Seven parallel replicates of each sample at each time point were prepared during this cell growth assay. Experiments were performed in triplicate for each group.

### 2.8 Statistical analysis

Data for the biomedical measurements were analyzed using one-way analysis of variance (ANOVA) for multiple comparisons with Tukey’s multiple comparison test. GraphPad Prism 6.0 software was utilized to analyze and perform the statistical significance of the assays, and differences at *p* < 0.05 were considered statistically significant.

## 3 Results and discussion

### 3.1 Chemical composition analysis of the pretreated WS

**FIGURE 1:**
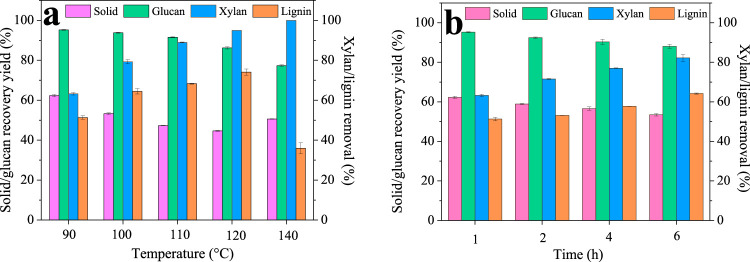
Chemical compositions of the pretreated WS at different temperatures **(A)** and times **(B)**.

Deep eutectic solvents have been used in recent years for successful biomass fractionation. In this study, a ChCl/BDO/AlCl_3_ system was proposed and investigated due to its mild pretreatment condition and excellent performance. First, we optimized the condition of the DES pretreatment, and the results are shown in Figure 1. The raw WS contains 34.25% cellulose, 16.51% xylan, and 22.70% lignin. It can be seen that even at the temperature of 90°C, only 62.27% ± 0.61% solid was recovered, indicating the excellent fractionation performance of our DES. The solid recovery further decreased gradually with increasing the pretreatment temperature, and finally, the lowest value of 44.68% ± 0.18% was observed at 120°C. However, the solid recovery yield increased to 50.61% ± 0.13% as the temperature reached 140°C. For each component, the xylan removal increased greatly with the increase in temperature, and finally, at 140°C, all of the xylan was degraded. Lignin was also depolymerized significantly during the pretreatment, and its removal increased gradually from 51.31% ± 0.90% (90°C) to the highest value of 74.10% ± 1.51% (120°C) with the increase in temperature. Similar to the solid recovery, the lignin removal decreased (35.96% ± 2.76%) as further increasing the temperature to 140°C, which is possibly due to the formation of pseudo-lignin and lignin condensation at high temperatures that precipitated back onto the substrate surface (Huang et al., 2022). The decrease of lignin removal at 140°C also explained the increased solid yield. In the case of glucan, it remained high within 90°C–110°C (95.24%–91.56%), indicating glucan is stable at lower temperatures. However, glucan recovery decreased to 86.21% ± 0.54% and 77.31% ± 0.44% at 120°C and 140°C. These results revealed that glucan may also degrade at high temperatures (Hossain et al., 2021).

Many colleagues have reported that lignin from low temperatures had high biocompatibility and low cytotoxicity, which is due to lignin’s structural changes at high temperatures (Figueiredo et al., 2018; Garrote et al., 2004). Therefore, to obtain high-quality lignin, we chose the low temperature of 90°C with reasonably high lignin removal of 51.31% for the following time investigation. It can be seen that the solid recovery only slightly decreased from 62.27% ± 0.61% to 53.43% ± 0.60% as the time was prolonged from 1 h to 4 h. In addition, xylan and lignin removal was found to increase with the pretreatment time and showed the highest value of 82.20% ± 1.71% and 64.20% ± 0.27% at 4 h. Glucan is very stable and only slightly decreased from 95.24% ± 0.18% to 88.07% ± 0.99%. It can be seen that compared to temperature, increasing time has a very limited effect on fractionation; thus, 1 h is likely to be sufficient for lignin isolation.

### 3.2 Analysis of the isolated lignin

After isolation using the DES, the lignin could be readily recovered using an antisolvent procedure. The recovery yield of the lignin is shown in Table 1. The lignin recovery yield was beyond 90%, indicating that almost all the isolated lignin can be recovered. In addition, sugar analysis revealed that only negligible sugars (<1%) existed in the recovered lignin, indicating its high purity.

**TABLE 1:** Lignin recovery yield and sugar content analysis.

Temp (°C)	Lignin recovery (%)	Glucose (%)	Xylose (%)	Arabinose (%)
L90	97.58	0.08	0	0
L100	96.46	0	0.05	0
L110	94.81	0.04	0.06	0
L120	98.32	0	0	0
L140	94.57	0.05	0.07	0

**FIGURE 2:**
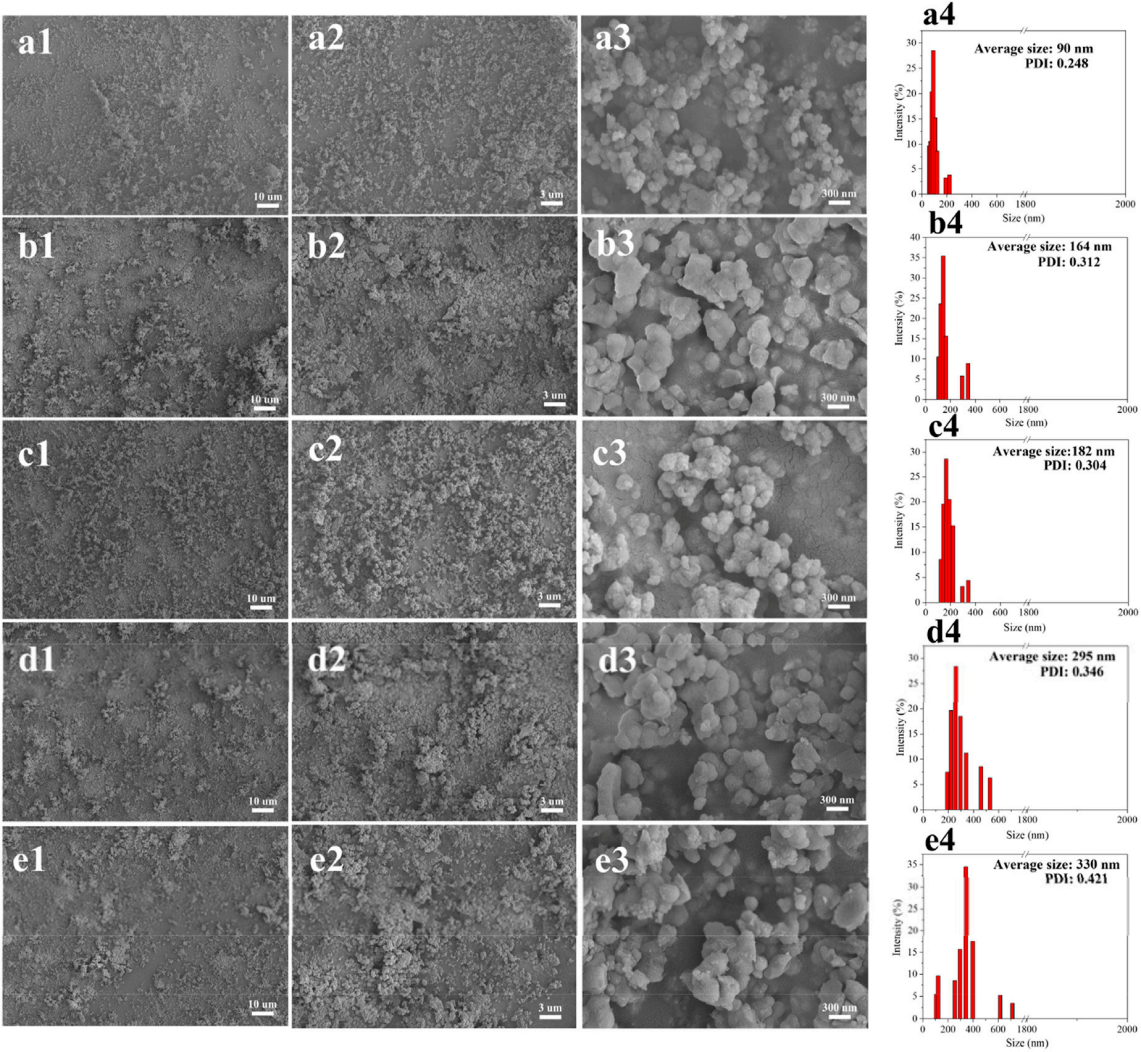
Morphology and size distribution of the regenerated LMSs at 90°C **(A1–A4)**, 100°C **(B1–B4)**, 110°C **(C1–C4)**, 120°C **(D1–D4),** and 140°C **(E1–E4)**.

**FIGURE 3:**
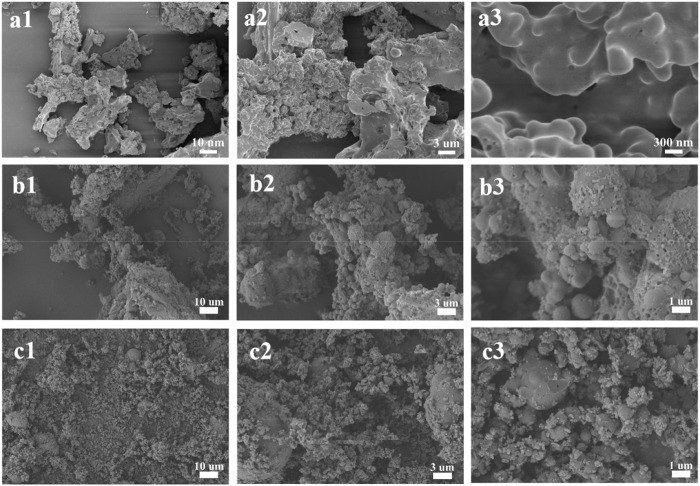
Morphology of CEL **(A1–A3)** and the lignin from ChCl/oxalic acid **(B1–B3)** and ChCl/lactic acid **(C1–C3)**.

Surprisingly, when we studied the morphology of the regenerated lignin using the FE-SEM, it can be seen that all the lignin presented a spherical shape, and the surface of the particles was quite neat and smooth (Figure 2), different from that of the native CEL (Figure 3). To make a comparison, we also conducted a pretreatment using common acid DES, such as ChCl/oxalic acid and ChCl/lactic acid. The results showed that the lignin from acid DES had an irregular shape (Figure 3). Uniquely, our BDO DES generated a lignin with sphere-like shape that can be an ideal material for biomedical applications. The diameter and size distribution of the LMSs were analyzed by a zeta sizer, and it was found that the average diameter enlarged with the increase in temperature, which ranged from 90 nm (90°C) to 330 nm (140°C). This is likely because, at higher temperatures, lignin can more readily suffer from condensation, which combines the nearby lignin molecules, forming large LMS particles. However, at the investigated conditions, all the LMSs are in the nano size range with a high yield that outperforms that of traditional LMS preparation methods (Alqahtani et al., 2019; Lievonen et al., 2016).

**FIGURE 4:**
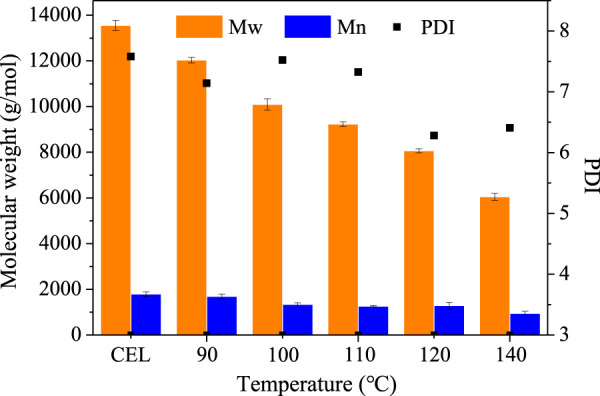
Molecular weight of the LMSs isolated at different temperatures.

The molecular weight of the regenerated lignins was measured by GPC, and the results are shown in Figure 4. The CEL has a molecular weight (MW) of 13,554 g/mmol and a number average molecular weight (Mn) of 1,788 g/mol with a polydispersity index (PDI) of 7.58. After pretreatment, both the Mw and Mn decreased with the increased temperature. For example, the MW of the lignin decreased from 13,554 ± 215 g/mmol to 12,034 ± 120 g/mmol (90°C), to 10,094 ± 249 g/mmol (100°C), to 9,231 ± 100 g/mmol (110°C), to 8,065 ± 90 g/mmol (1200°C), and to 6,049 ± 148 g/mol (140°C). Also, the Mn decreased slightly from 1,788 ± 97 g/mol (CEL) to 944 ± 93 g/mol (140°C). It should be noted that the lignin molecular weight of this study is much higher than that of other DES lignins, which normally had a Mw < 2000 g/mol. As is well known, the molecular weight of lignin is strongly associated with the structure integrity in which the high Mw lignin had a more intact structure. This indicated that the lignin from our BDO pretreatment may have a more intact structure, as discussed in the following section.

### 3.3 Structure analysis of the lignin microspheres

**FIGURE 5:**
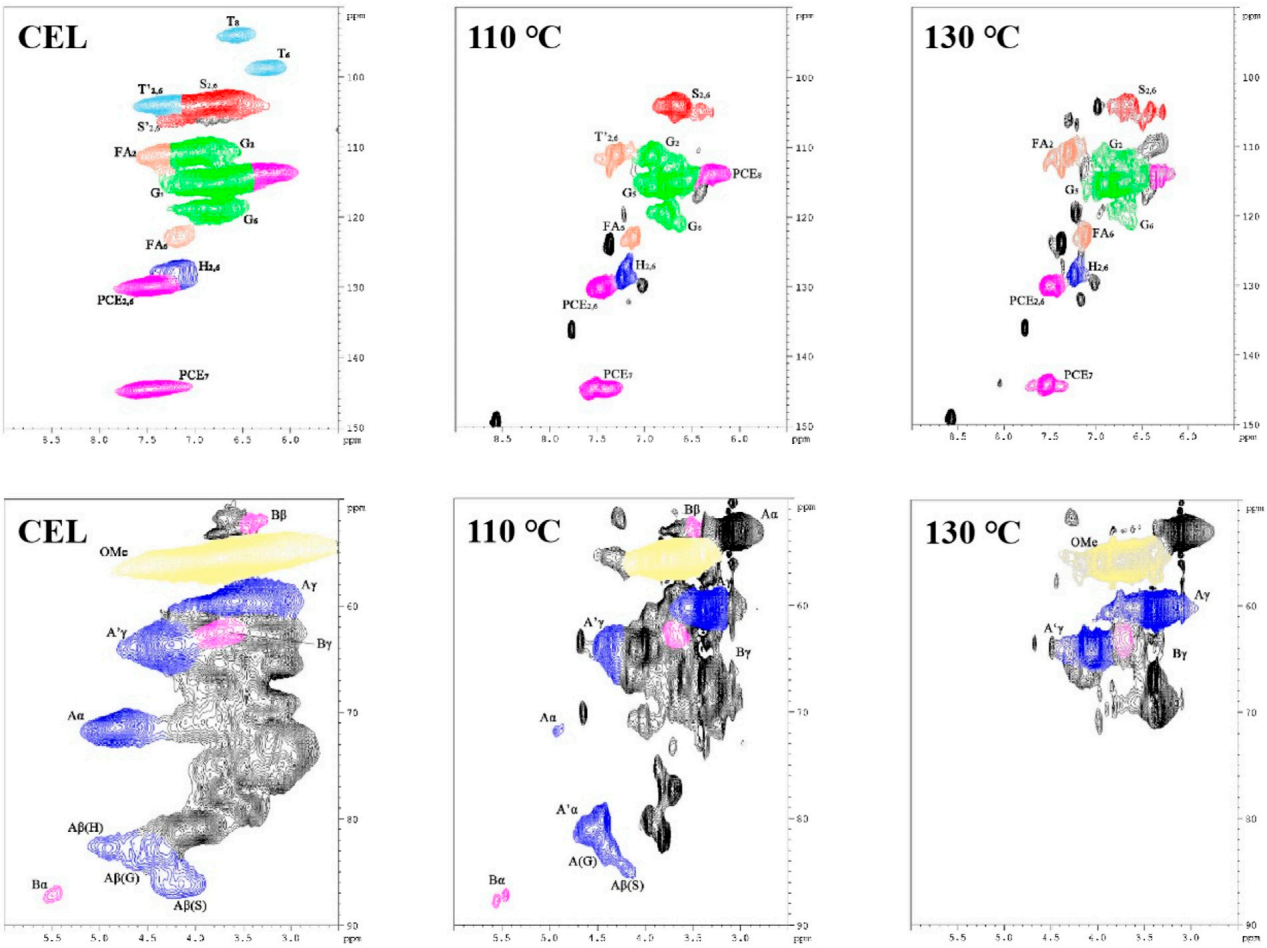
HSQC NMR spectra of the WS CEL and the LMSs (upper row shows the aromatic region; lower row shows the side-chain region).

The chemical structure changes of the lignin before and after isolation were investigated using 2D HSQC NMR, and the results are shown in Figure 5. The signal assignment was discussed in previous publications (Cai et al., 2020; Sosa et al., 2020).

In the aromatic region, typical lignin structure characteristics of herbaceous biomass were seen for WS CEL, including S, G, and H units and the associated T and *p*CE signals. In detail, the S_2,6_ signal is located at 103.7/6.72 ppm, while those of G_2_, G_5,_ and G_6_ are found at 110.8/6.97 ppm, 115.3/6.77 ppm, and 118.9/6.80 ppm. The H_2,6_ signal is located at 128.0/7.20 ppm. In addition, the signals of other grass substructures, such as tricin (T), ferulic acid (FA), and *p*-coumarate (*p*CE), are also observed. After extraction with the DES, the regenerated lignin showed a quite different profile, in which all the basic units weakened substantially, and the T signals even disappeared. In addition, significant lignin condensation is observed from the spectra for the S, G, and H units as their chemical shift changed to some extent, especially at 130°C. As shown in Table 2, raw WS CEL contains 34.47% S, 57.42% G, and 8.11% H with an S/G ratio of 0.60. In addition, 21.04% FA and 55.57% *p*CE exist in the CEL, indicating the abundance of the lignin–carbohydrate complex in WS lignin. For the regenerated lignin spheres, the FA content significantly decreased to 9.06% at 110°C, and finally, at 130°C, it disappeared. These results are consistent with previous reports that the lignin–carbohydrate complex linkages are liable to suffer from cleavage during DES isolation (Chen et al., 2022). The amount of *p*CE remained almost constant. In addition, it can be seen that 5.17% condensed S unit and 13.72% condensed G unit were observed for 110°C lignin, which both increased at 130°C.

**TABLE 2:** Quantification of CEL and the regenerated LMSs from HSQC NMR spectra (presented with units per 100 Ar).

	S	Con S	G	Con G	H	Con H	S/G	FA	pCE	β-O-4	β-O-4′	β-β
CEL	34.47	0	57.42	0	8.11	0	0.60	21.04	55.57	48.74	0	1.48
110	27.29	5.17	37.04	16.72	10.61	3.18	0.60	9.06	56.53	3.56	34.26	0
130	19.59	8.52	26.99	13.72	13.72	5.31	0.53	0	48.19	0	0	0

In the aliphatic region, signals of β-O-4 and β-β are observed, indicating these two linkages are the dominant bonds between lignin units. Especially, signals of A_α_, A_β_, and A_γ_ are located at 71.54/4.84 ppm, 86.17/4.15 ppm (A_β_ of S), 82.59/4.42 ppm (A_β_ of G), 82.68/4.92 ppm (A_β_ of H), and 59.56/3.59 ppm (A_γ_). In addition, β-β signals are found at 87.12/5.49 ppm (B_α_), 52.25/3.35 ppm (B_β_), and 62.61/3.80 ppm (B_γ_). All these signals became weak for the lignins at regenerated at 110°C. From the semiquantitative data, we can also see that only 3.56% of the β-O-4 bond was preserved at 110°C, and it was not detected at 130°C (Table 2). However, a new signal at 81.08/4.55 ppm appeared after the lignin isolation. This new signal was assigned to the β-O-4 derivative and is termed β-O-4’ (Cheng et al., 2023b). During the lignin extraction by the DES, the α position of the β-O-4 structure was first cationized by the departure of the hydroxyl groups. In normal pretreatment, this reaction will inevitably cause the lignin β-O-4 cleavage and further induce the lignin condensation reaction. However, in our pretreatment, the BDO as a carbocation scavenger attacked the α position of the β-O-4 bond and quenched the carbocation, which protected the bond from cleavage and yielded an etherified β-O-4 structure (β-O-4′) (Cheng et al., 2022). With this effect, it can be found that as much as 34.26% of the β-O-4 bond was transformed into β-O-4′, with a total aryl ether bond of 37.82%. This result indicated that our DES could well preserve the β-O-4 bond, which prevents the lignin from condensing and may affect its biological effect. However, as the temperature increased further to 130°C, all the linkages were broken significantly.

**FIGURE 6:**
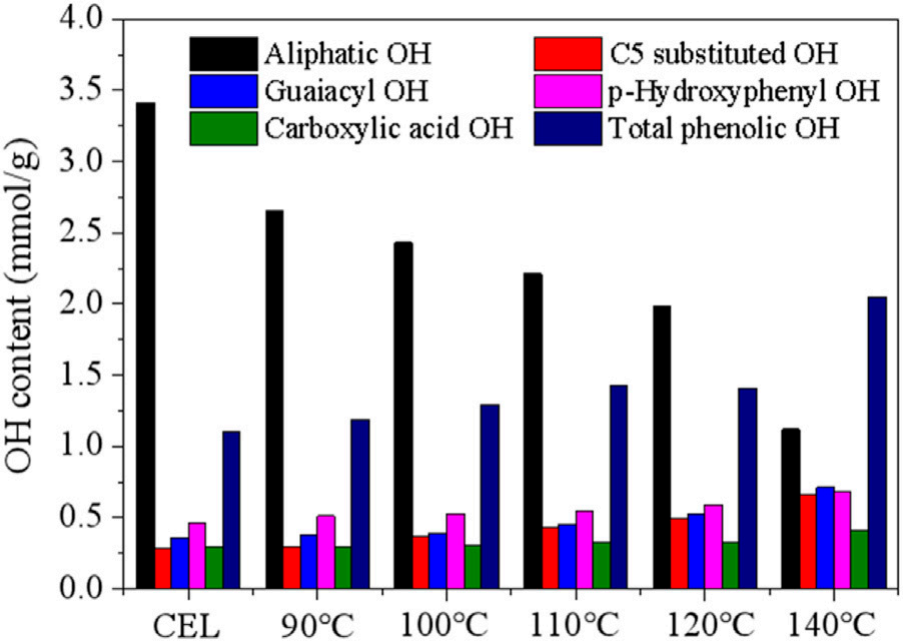
Hydroxyl groups of the raw WS CEL and the recovered LMSs determined by ^31^P NMR.

^31^P NMR was then performed to measure the various hydroxyl groups in the CEL and the recovered LMSs, and the results are shown in Figure 6. As can be seen, the raw WS CEL has 3.41 mmol/g aliphatic OH, accounting for 71.04% of the total hydroxyl groups. The aliphatic OH gradually decreased as the pretreatment temperature increased from 90°C to 140°C, which is mainly ascribed to the departure of the OH in the lignin’s Cγ position. The syringyl and condensed OH signals in the NMR spectra overlap; thus, they were combined into C5-substituted OH. In the raw CEL, C5-substituted OH indicated the S-OH, as no lignin was condensed in raw WS, possessing a content of 0.28 mmol/g. Its content remained stable at 90°C and 100°C, which were 0.30 mmol/g and 0.37 mmol/g, respectively, indicating lignin condensation is very limited at low temperatures. While as further temperature increases, the C5-substituted OH increased greatly and reached 0.66 mmol/g at 140°C. The G- and H-OH followed a similar trend to that of the C5 substituted OH. The total phenolic OH was 1.1 mmol/g in raw CEL and increased gradually to 1.19 mmol/g, 1.29 mmol/g, 1.43 mmol/g, 1.61 mmol/g, and 2.05 mmol/g due to lignin depolymerization. The increased phenolic OH also explained the enhanced antioxidation of the lignins, as demonstrated later.

The carboxylic acid OH increased slightly from 0.29 mmol/g (raw CEL) to 0.41 mmol/g (140°C) due to the partial lignin oxidation at high temperatures. Previous publications indicated that the hydrophobicity of lignin, which is strongly related to its phenolic OH content and condensation degree, is the driving force for the LMS formation, and the lignin with high phenolic OH likely tended to yield small LMSs. However, in this study, the LMSs at high temperatures are larger. This result indicated that other factors, such as the lignin molecular weight and lignin condensation degree, may play a more important role in the formation of LMSs.

### 3.4 Antioxidation property of the LMSs

**FIGURE 7:**
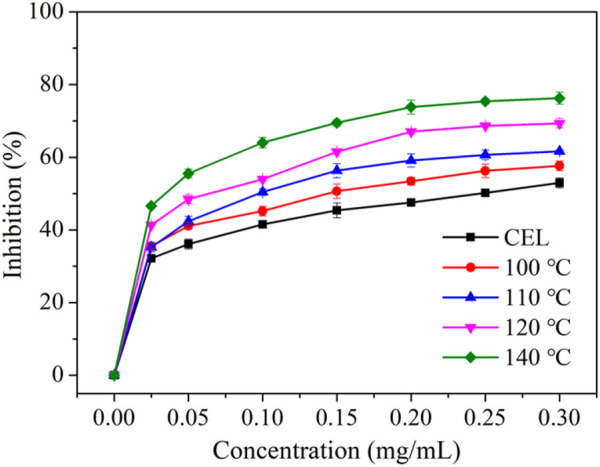
The antioxidation property toward DPPH of raw WS CEL and the LMSs generated at different temperatures.

Lignin has been reported to have excellent antioxidation properties and is thus considered to be an ideal candidate for application in the biomedical field. The antioxidation property of the LMSs was measured by testing its capacity to eliminate the free radical DPPH, and the results are provided in Figure 7. It can be seen that the antioxidation increased with the rise of lignin concentration, especially for those with >0.20 mg/mL lignin addition, which possessed a scavenging activity of >50%. The amount of antioxidation remained almost unchanged as the lignin addition was further increased. A clear trend was found that the antioxidation increased when using the LMSs from higher temperatures, which presented the highest values of 52.99% (CEL), 57.65% (100°C), 61.66% (110°C), 69.33% (120°C), and 76.26% (140°C). This result is ascribed to the structural changes of the lignin during the isolation. A previous study demonstrated that the content of S and G, as well as the condensation section, may affect lignin oxidation. From our results, we can conclude that the decrease in S/G ratio and also the increase in condensed structure likely enhanced the antioxidation of the LMSs, as the demethoxylation increased at higher temperatures. This result is in contrast to those of a previous study in which it was found the antioxidation increased with the increase of methoxy substitute (Lourençon et al., 2021). One possible explanation of this result may be that the molecule weight of the LMSs decreased with increasing temperature, and the small lignin moiety may expose more phenolic hydroxyl groups and thus show better antioxidation properties. In addition, the change in interlinkages, as well as the decrease in FA and *p*CE, may also influence the antioxidation. Previous studies indicated that lignin antioxidation is positively correlated with the contents of FA and *p*CE (Dong et al., 2019; Garrote et al., 2004), while in this study, we observed a different result. An explanation is that the antioxidation of the lignin is strongly associated with its phenolic OH, which is abundant in the lignins from high temperatures, while the FA and *p*CE tend to degrade at higher temperatures. The IC50 value was found to decrease from 0.46 mg/mL to 0.06 mg/mL as the temperature increased, further confirming this result.

### 3.5 Biomedical test

**FIGURE 8:**
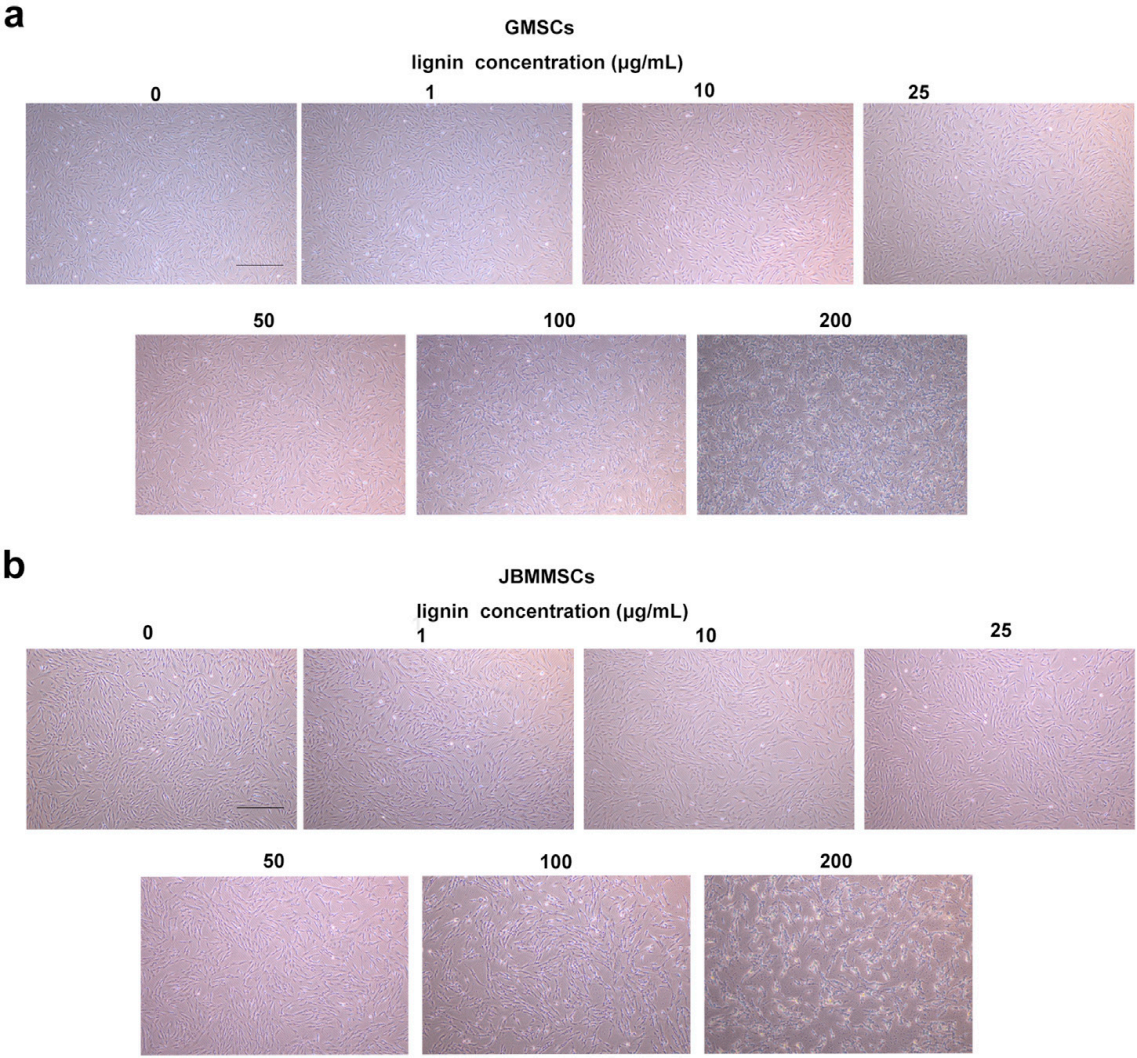
Microphotographs of GMSCs **(A)** and JBMMSCs **(B)** treated with various concentrations of lignin for 24 h (the scale bar in these pictures is 400 μm).

To evaluate the potential of our LMSs in the biomedical application, their biocompatibility was tested by cultivating them with GMSCs and JBMMSCs. These cells were isolated from clinically discarded gingival and jaw bone marrow tissues. They were first cultured in the media for proliferation until obtaining the desired amounts. We then cultivated the cells with different concentrations of lignin (isolated at 90°C) ranging from 0 to 200 μg/mL (Figure 8). It can be seen that at an LMS addition <25 μg/mL, both GMSCs and JBMMSCs remained in good growth condition with almost no difference in morphology and density. These results suggest our LMSs showed excellent biocompatibility in certain additions. Further increasing the LMS concentration to 100 μg/mL, an obvious decrease of cell adherence can be observed, and finally, at 200 μg/mL, significant cell apoptosis was found, indicating high LMS usage inhibited the cell growth of GMSCs and JBMMSCs.

**FIGURE 9:**
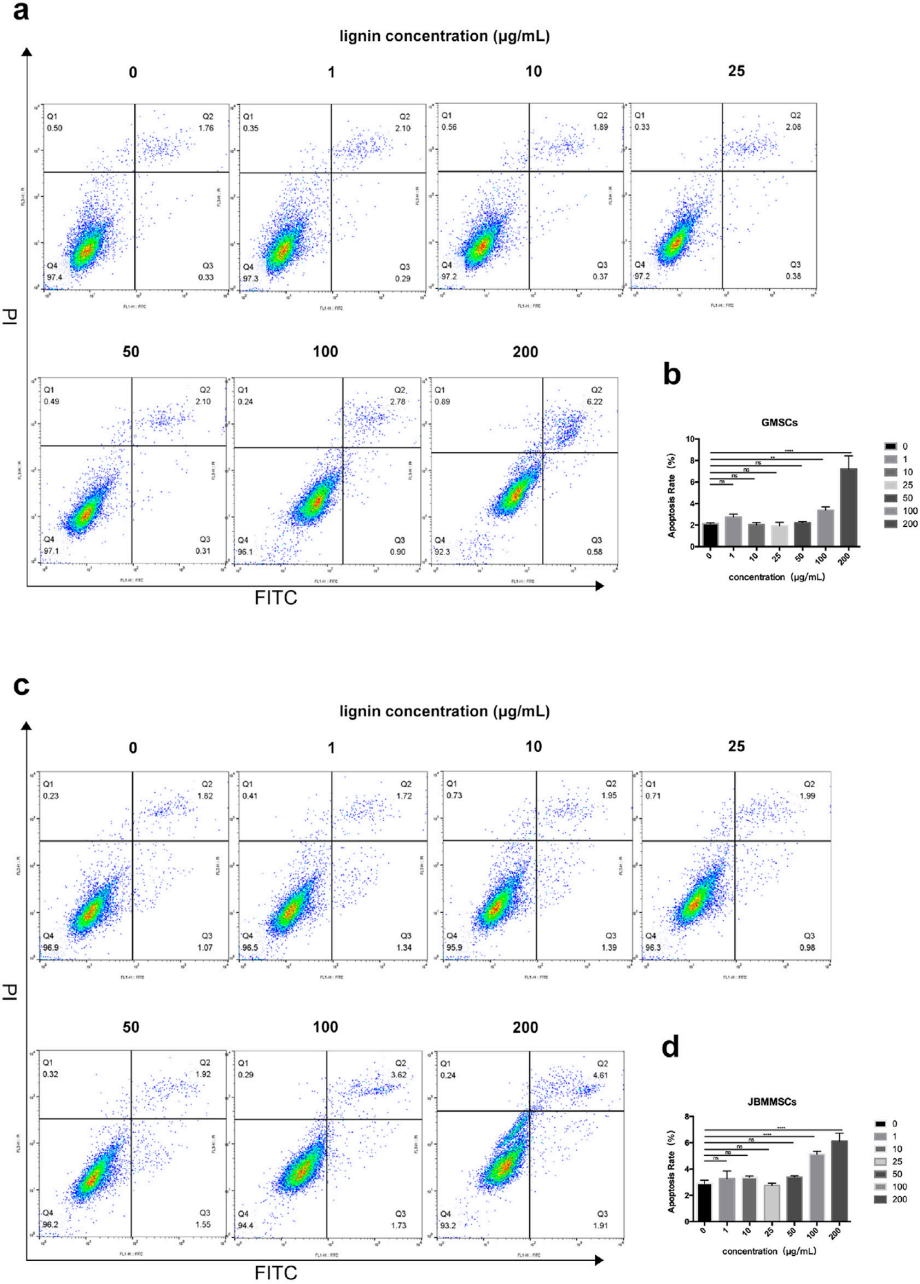
Effects of various lignin concentrations on the apoptosis of GMSCs **(A, B)** and JBMMSCs **(C, D)** by flow cytometry analysis for 24 h. ** indicates *p* < 0.01. *** indicates *p* < 0.001 (one-way analysis of variance). The results represent mean ± SD. ns, no statistically significant difference (*p* > 0.05); group, n = 5.

The above result indicated the good antioxidation and biocompatibility of our DES lignin. To further investigate the potential application in the oral area, its cytotoxicity was assessed for 24 h, as shown in Figure 9. As can be seen, at a lignin usage of 1–50 μg/mL, very limited cytotoxicity for GMSCs was observed as the apoptosis was all below 3%, almost the same as that of the control with no lignin addition (*p* > 0.05), indicating its excellent biocompatibility at a certain concentration. Even at a lignin usage of 100 μg/mL, the apoptosis rate was only slightly increased to 3.3%. Furthermore, at 200 μg/mL lignin addition, the apoptosis rate significantly enhanced and reached 7.1%. A similar phenomenon was also observed for JBMMSCs, which yielded a low apoptosis rate even at a 200 μg/mL lignin concentration.

**FIGURE 10:**
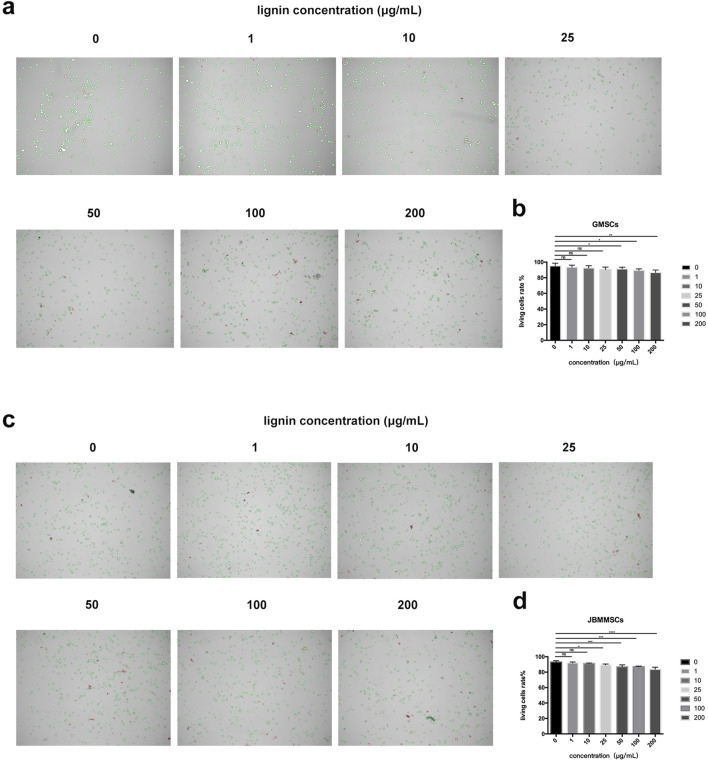
Effects of various lignin concentrations on the living status of GMSCs **(A, B)** and JBMMSCs **(C, D)** with Trypan blue stain for 48 h. * indicates *p* < 0.05. ** indicates *p* < 0.01. *** indicates *p* < 0.001 (one-way analysis of variance). The results represent mean ± SD. ns, no statistically significant difference (*p* > 0.05); group, n = 5.

To confirm the biocompatibility of our LMSs, we cultured the cells with LMSs for 48 h, and then the amount of living cells was quantitatively determined by the Trypan blue staining. During the Trypan blue (TB) staining, the dye cannot enter the living cells, which present a green color, while the TB molecule can enter the cell membrane of dead cells and stain the cells, which show a red color. As shown in Figure 10, for treatment with low lignin addition, the ratio of blue to red cells was relatively stable, in which the living cell ratios are 93.5% (1 μg/mL), 92.5% (10 μg/mL), 90.5% (25 μg/mL), and 91.5% (50 μg/mL), close to that of the control test (95.25%) (*p* > 0.05). As the lignin increased > 100 μg/mL, the number of dead cells increased gradually, and finally, at 200 μg/mL lignin, the live cell ratio decreased to 86.5%. As for the JBMMSCs, the cytotoxicity was even stronger as an obvious increase in dead cells (live cell ratio of 87%) was observed for a 50 μg/mL lignin addition (*p* < 0.01).

**FIGURE 11:**
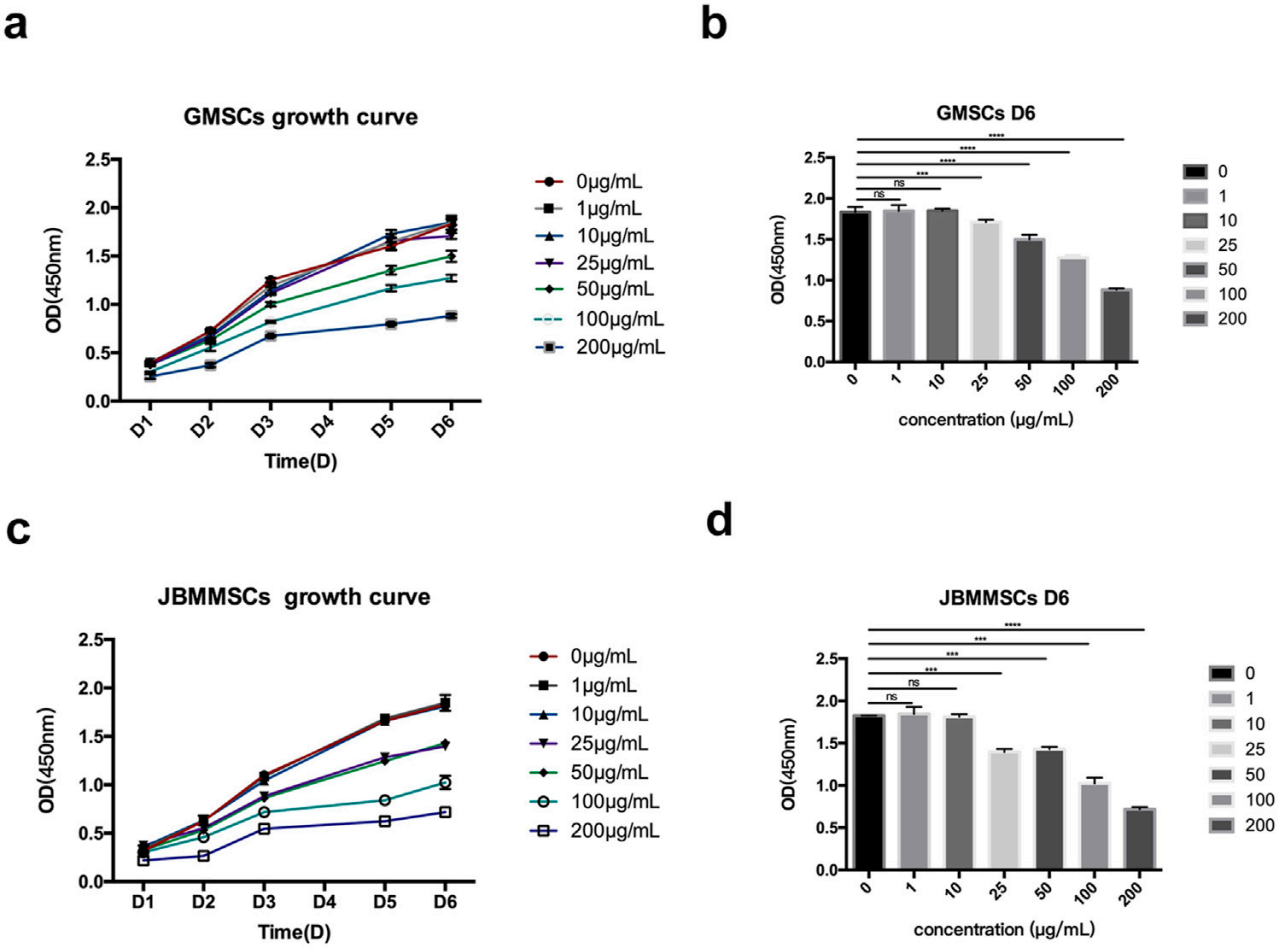
Effects of various lignin concentrations on the growth of GMSCs **(A, B)** and JBMMSCs **(C, D)** according to CCK-8 for 6 days. *** indicates *p* < 0.001 (one-way analysis of variance). The results represent mean ± SD; ns, no statistically significant difference (*p* > 0.05); group, n = 7.

Our LMSs were proven to have good antioxidation and biocompatibility at a reasonable concentration. Many publications also reported that lignin has low cytotoxicity due to antioxidation. Therefore, the *in vitro* cytotoxicity of the LMSs toward GMSCs and JBMMSCs was measured by the CCK-8 method for a longer time by observing the proliferation of cells with the LMSs. The growth curves were drawn according to the CCK-8 analysis after 6 days of proliferation with lignin (0 μg/mL, 1 μg/mL, 10 μg/mL, 25 μg/mL, 50 μg/mL, 100 μg/mL, and 200 μg/mL) (Figures 11A, B). As shown, the cytotoxicity was also dependent on the amount of added lignin. At a low amount of 1 μg/mL and 10 μg/mL, the OD value of both GMSCs and JBMMSCs was very close to the control without lignin, which indicated their low cytotoxicity at these concentrations. However, as the concentration of lignin increased (≥25 μg/mL), the proliferation ability of cells decreased. Both GMSCs and JBMMSCs with lignin (≥25 μg/mL) proliferated worse than the control run (*p* < 0.001) (Figures 11C, D). In addition, JBMMSCs were more sensitive to changes in lignin concentration than GMSCs. A similar observation was also reported by Zheng et al. in work culturing kraft lignin with chondrocyte cells (Zheng et al., 2021). In another work, the authors found that the cytotoxicity of lignin is related to the chemical structure in which lignin has more S units and more phenolic OH exhibited stronger cytotoxicity (Wang et al., 2021). In this study, we isolated nanosized LMSs using an effective method and measured their biocompatibility and cytotoxicity. More *in vivo* tests and studies of lignin’s working mechanism are ongoing and will be reported in later work.

## 4 Conclusion

This study proposed a BDO DES method that could effectively extract lignin nanospheres from wheat straw. The lignin spheres all presented a spherical shape with a diameter of 90–330 nm regulated by changing the isolation conditions. Structure analysis indicated the lignin spheres had a relatively intact molecular structure with a high β-O-4 bond. Antioxidation data showed that the LMSs all had good antioxidation properties, and the LMSs from high temperatures tended to perform better in scavenging DPPH. Biomedical tests indicated that these LMSs at certain usage (<25 μg/mL) had good biocompatibility toward cells from oral soft and hard tissues (GMSCs and JBMMSCs), which could be a new promising material for the treatment of oral diseases. Overall, this study demonstrated the potential of the new kind of LMS in oral tissue regeneration, whether directly used as a filler or as an adjuvant for the fabrication of scaffolds.

## Data Availability

The original contributions presented in the study are included in the article/supplementary material; further inquiries can be directed to the corresponding authors.
